# Migration rules: tumours are conglomerates of self-metastases

**DOI:** 10.1038/sj.bjc.6605071

**Published:** 2009-05-19

**Authors:** H Enderling, L Hlatky, P Hahnfeldt

**Affiliations:** 1 Center of Cancer Systems Biology, Caritas St. Elizabeth's Medical Center, Tufts University School of Medicine, 736 Cambridge Street, Boston, MA 02135, USA

**Keywords:** agent-based model, tumour progression, self-metastases, cancer stem cells, migration

## Abstract

Tumours are heterogeneous populations composed of different cells types: stem cells with the capacity for self-renewal and more differentiated cells lacking such ability. The overall growth behaviour of a developing neoplasm is determined largely by the combined kinetic interactions of these cells. By tracking the fate of individual cancer cells using agent-based methods *in silico*, we apply basic rules for cell proliferation, migration and cell death to show how these kinetic parameters interact to control, and perhaps dictate defining spatial and temporal tumour growth dynamics in tumour development. When the migration rate is small, a single cancer stem cell can only generate a small, self-limited clone because of the finite life span of progeny and spatial constraints. By contrast, a high migration rate can break this equilibrium, seeding new clones at sites outside the expanse of older clones. In this manner, the tumour continually ‘self-metastasises’. Counterintuitively, when the proliferation capacity is low and the rate of cell death is high, tumour growth is accelerated because of the freeing up of space for self-metastatic expansion. Changes to proliferation and cell death that increase the rate at which cells migrate benefit tumour growth as a whole. The dominating influence of migration on tumour growth leads to unexpected dependencies of tumour growth on proliferation capacity and cell death. These dependencies stand to inform standard therapeutic approaches, which anticipate a positive response to cell killing and mitotic arrest.

Beyond the genetic alterations known to advance carcinogenesis in single cells, the overall phenotype of a developing neoplasm is determined largely at the population level. This is because evolutionary competition among clones comprising a neoplasm will assure that only certain of these thrive to define the tumour. Even after a tumour is established, population-level bottlenecks, for example, the failure to acquire angiogenic and invasive competence, may determine whether a fully malignant tumour ever rises to clinical detection. A tumour can lie dormant below clinical detection for years (Nielsen *et al*, 1987; Black and Welch, 1993), limited by competition for nutrients (Folkman, 1971; Hahnfeldt *et al*, 1999; Naumov *et al*, 2006; Abdollahi *et al*, 2007). Only tumours that successfully surmount or circumvent all such population-level constraints will go on to grow. Accordingly, population-level kinetics are expected to augment cell-level genetics in driving tumour progression.

One population-level factor limiting tumour progression is the space to grow (Brú *et al*, 2003; Pardal *et al*, 2003; Drasdo and Höhme, 2005; Chao *et al*, 2008). The nature of this limitation has been debated, given the observation that tumour cells can push their neighbours under certain circumstances. However, as studied by Brú *et al* (2003) and confirmed by Drasdo and Höhme (2005), this ability is confined to a thin layer of cells near the surface of tumours and even to a thin ring of cells at the outer edge of well-nourished confluent tumour cell populations *in vitro*. Cells deeper in the tumours are limited by the surrounding cell mass. The transition of exponential to linear growth in tumour diameter (corresponding to d*V*/d*t*=*kV*^2/3^), noted when the tumour spheroids reach about 200 *μ*m in diameter (Drasdo and Höhme, 2005) suggests that the thickness of this zone for solid tumours is just 100 *μ*m or so.

A second key factor proving to be important is proliferation capacity. According to the cancer stem cell hypothesis that has now garnered substantial support (Al-Hajj *et al*, 2003; Singh *et al*, 2003; Boman and Huang, 2008; Kakarala and Wicha, 2008), tumour cells do not all divide indefinitely, as tacitly assumed in the classic carcinogenesis paradigm (Hanahan and Weinberg, 2000). Instead, only a small fraction of cells have the capacity for limitless division and self-renewal (the so-called ‘cancer stem cells’). The rest are able to divide only a certain number of times before they deplete their generational capacity. Supporting the theory, small cancer stem cell populations are strongly implicated in leukaemia (Furth and Kahn, 1937; Kavalerchik *et al*, 2008) and tumour-initiating side populations have also recently been identified in solid tumours of the breast, colon and prostate, for example (Al-Hajj *et al*, 2003; Boman and Huang, 2008; Kakarala and Wicha, 2008; Maitland and Collins, 2008; Price *et al*, 2008).

Taking these basic population-level features into account, we designed an agent-based computer model of cancer cell dynamics operative throughout tumour development (see Anderson *et al* (2007) and Deutsch and Dormann, (2005) for similar approaches). Tracking three cell-level kinetics – cell death, proliferation capacity and migration rate – in a simulated cancer cell population composed of cancer stem and non-stem cells, we show how they interact to produce emergent population-level effects. Owing to the strong limitation on the ability of tumour cells to push on other tumour cells, for example, clustering of progeny around the tumour is seen to cause cell crowding and consequent self-inhibition of the tumour as a whole if most cells have a limited generational life span. If instead the tumour cells tend to separate, distinct tumour clusters arise and grow to a larger total size because of a decreased self-inhibition resulting from a lower average cell density. A larger tumour is the result.

In this way, metastasis and tumour growth are interrelated. It is understood that metastases arise from cancer stem cells that escape the primary cell cluster to seed independent clusters elsewhere. It has been proposed that some of these cells might either expand into, or circulate and reappear at the edge of the primary site, fuelling local growth by ‘self-metastasis’ (Norton, 2005; Norton and Massague, 2006). We show how the combined actions of migration, limited proliferation and cell death combine counterintuitively to advance tumour development, and how cell migration, in particular, might drive self-metastatic growth through the seeding of new tumour clones directly in the tumour vicinity.

Interestingly, these mechanisms reproduce population-level behaviours often attributed to higher-order biological processes. As cases in point, the impact of cell crowding and the importance of migration have earlier been analysed in a model of vascular tumour growth (Betteridge *et al*, 2006). The authors reported that with increasing tumour carrying capacity because of increased vasculature, significant cell movement increases the rate of tumour growth and invasion. In another study, an agent-based approach was used to study emerging patterns in tumour systems (Mansury *et al*, 2002). In this model, heterogeneous nutrient concentrations and directed cell migration towards nutrient attractors were determined to be necessary for cluster formation. With our simpler model, however, we could reproduce this finding without a structured environment. This leads us to propose that some considerations of higher-order biological mechanisms in tumour modelling, while providing new insights into tumour growth control, may be ascribing higher-order mechanisms to tumour growth characteristics that can be explained by more basic cell-level kinetics.

## Methods

**Figure 1:**
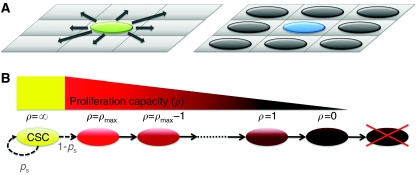
Cell migration and proliferation. Tumour cells come to occupy adjacent lattice points by two means, migration and proliferation. (**A**) A cell can randomly migrate to one of the eight adjacent lattice points, if one is available, vacating the original lattice point, or it can proliferate, with a daughter randomly occupying an adjacent lattice point, if one is available (green cell, left panel). If all the eight lattice points contain a cell, a cell attempting to migrate will do nothing, and a cell attempting to divide will become quiescent instead (blue cell, right panel). (**B**) Proliferation of a cell is ultimately limited by its proliferation capacity. Cancer stem cells (CSCs) have unlimited replicative potential and self-renewing ability. With probability, *p*_s_, a new CSC is produced, and with probability, 1-*p*_s_, a non-stem progeny cancer cell is produced that, with each division, loses proliferation capacity until proliferation is exhausted and the cell dies.

An agent-based model is employed that treats cells as ‘agents’ that follow a specific set of rules influenced by the local environment. The evolution of the population, starting from a single cancer stem cell, is tracked by following cell actions in a computational domain. The computational domain consists of a 350 × 350 array of square lattice points, each measuring 10 × 10 *μ*m. Each lattice point can accommodate at most one cell at any time. Each cell can proliferate after 1 day, provided the daughter cell can enter an empty adjacent lattice point. An adjacent lattice point is here defined as one of the eight lattice points surrounding a given lattice point (Figure 1A). We start the simulations with a single cancer stem cell with unlimited replicative potential *ρ*=∞ placed at the centre of the domain (the lattice point 175 rows down, 175 columns across). To accommodate the known limitation on cancer cell growth imposed by space constraints (Brú *et al*, 2003; Drasdo and Höhme, 2005; Galle *et al*, 2009), if all eight adjacent lattice points are occupied, a cell is considered inhibited and sent into quiescence until neighbouring space becomes available. Similar computational assumptions were made in a recent *in silico* model of opportunistic preneoplastic lesion growth driven by the death of adjacent normal stem cells (Chao *et al*, 2008). How these assumptions are consistent with a tumour expanding into normal tissue has been argued based on a principle of compactness (Norton, 2005). Considering normal breast tissue to be only 75% compact compared with 100% for tumours (logical estimates based on mammography), a tumour would invade the less dense normal tissue, but not vice versa. At the same time, the tumour would be limited with respect to invasion within its own 100% compact mass because of the strongly limited ability of tumour cells to push themselves, as discussed earlier.

In light of evidence for a small cancer stem cell fraction in the tumour mass, we further assume that stem cells either divide symmetrically to produce another stem cell with a small probability, *p*_s_, or divide asymmetrically with probability, 1–*p*_s_, to produce a stem cell and a non-stem progeny cell with proliferation capacity *ρ*=*ρ*_max_. A default value, *p*_s_=1%, was selected as representative, reflecting (1) the order of magnitude of stem cell frequency observed in solid tumours (Visvader and Lindeman, 2008), and, as we later discuss, (2) the surprising insensitivity of the basic tumour growth characteristics for *p*_s_ values ranging from 1 to 50% and beyond. If non-stem cells divide, their proliferation capacity *ρ* decreases by 1, and the daughter cells inherit the new *ρ* (Figure 1B). Eventually, these cells exhaust their proliferative capacity and cease dividing. The disposition of such cells has been the subject of a number of papers. In a study comparing breast tumour stem and non-stem cells, Li *et al* (2007) measured telomerase activity and found that cancer stem cells show a higher level of telomerase enzyme activity than non-stem cancer cells. Zhang *et al* (1999) showed that telomerase inhibition in tumour cells triggers apoptotic cell death. Taken together, the results suggest that non-stem tumour cells mostly undergo cell death after exhaustion of generational capacity. Accordingly, we remove cells from the domain when their proliferation capacity is exhausted, that is, *ρ*=0.

**Figure 2:**
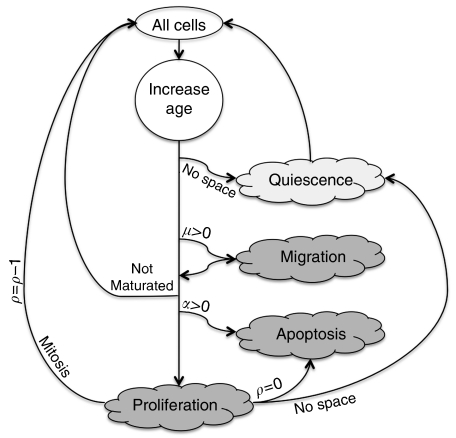
Cell life cycle scheme. At each time step, the cell age increases. The cell can migrate, and if maturation age is reached the cell can proliferate, if space is available, or enter quiescence otherwise. If proliferation capacity is exhausted the cell dies; otherwise, it produces a daughter cell.

With equal likelihoods, cells can migrate randomly into one of the adjacent available lattice points (a typical rate being 0.00635 mm h^−1^ or about *μ*=15 cell widths per day (Maini *et al*, 2004) or remain stationary. At each time of potential proliferation, we further assume that cancer cell death occurs spontaneously and randomly among non-stem cells with probability *α*. Different sources of spontaneous cancer cell death exist; for instance, spontaneous cell apoptosis (Meggiato *et al*, 2000) has been estimated to be in the order of 1–25% in breast cancer (Ehemann *et al*, 2003). A schematic of the cell life cycle scheme in our computational model is shown in Figure 2. We run each stochastic simulation for *t*=20 × 365 days, that is, 20 years, unless the domain becomes confluent sooner.

## Results

**Figure 3:**
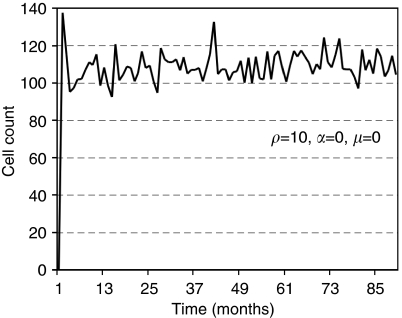
Tumour population dynamics for *t*=0–90 months with low progeny proliferation capacity (*ρ*=10), and disabled migration and spontaneous cell death (i.e., *α*=*μ*=0). The tumour size oscillates because of dying cells at the outer rim, which get replaced by cells from the tumour core that were previously quiescent. The initiating single cancer stem cell is not liberated sufficiently to divide symmetrically, resulting in a pseudo-steady-state tumour size at around 110 cells.

**Figure 4:**
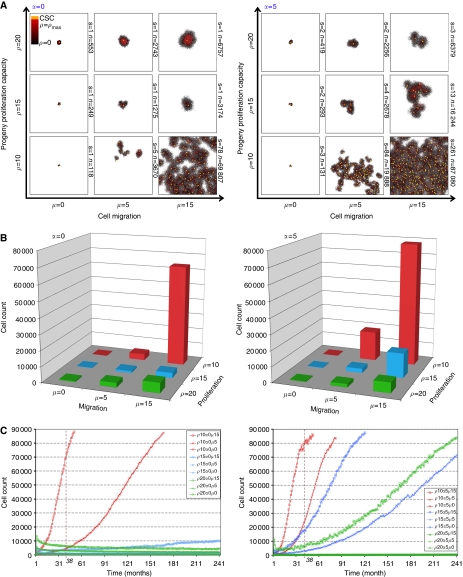
Tumour growth dynamics over time for representative parameters. For progeny proliferation capacities, *ρ*=(10,15,20), migration rates, *μ*=(0,5,15) and spontaneous cell death rates, *α*=(0%, 5%), per day, tumour growths are simulated. (**A**) Representative simulation results after *t*=38 months. Without cell death and migration (*α*=*μ*=0), no malignant tumour can form. With increasing migration rate, the tumours grow bigger, but for tumours with low progeny proliferation capacity (*ρ*_max_=10) symmetric stem cell divisions occur frequently, allowing for tumour growth expansion. Increasing the spontaneous death rate to *α*=5% liberates the stem cells to develop multiple clusters of tumour cells, if the migration rate is sufficiently high. S: number of cancer stem cells; n: number of cancer cells. (**B**) Average cell counts. Average cell counts are shown at *t*=38 months for different migration rates and proliferation capacities for 40 simulations each, without (*α*=0) and with (*α*=5) spontaneous cell death. (**C**) Average tumour growth curves for all parameters for *t*=240 months (i.e., 20 years). The vertical dotted lines show the tumour cell count at *t*=38 months.

We use the agent-based model to simulate tumour development dynamics with various cellular migration potentials (i.e., *μ*=0, 5 and 15 cell widths per day), non-stem cancer cell proliferation capacities (i.e., *ρ*_max_=10, 15, 20 divisions), and spontaneous cell death rates (*α*=0 and 5% per day). When a single cancer stem cell is placed in the centre of the domain and cellular migration and spontaneous cell death are disabled (*μ*=0 and *α*=0%, respectively), proliferation and cell death after proliferation capacity is exhausted are the defining tumour dynamics. The first daughter cell is produced after *t*=1 day. Both cells, without migration, occupy adjacent spaces, and their next generation progeny are placed next to their respective parents. Therefore, on a two-dimensional lattice, the cancer stem cell can initially produce at most eight daughter cells (if none of the first daughter's progeny randomly places a daughter cell next to the stem cell), and in the least favourable case only three daughter cells (if all the first daughter's progeny are randomly positioned next to the stem cell), before it becomes space-inhibited and thus quiescent. The cancer stem cell divides symmetrically with probability, *p*_s_=0.01, to generate a sister cancer stem cell. Even if a second cancer stem cell arises before all neighbouring lattice points are occupied by progeny to the first stem cell, that second stem cell would also produce daughter cells and, thus, likely be forced to remain resting until exposed to space again. In this scenario, it is clear that the tumour growth dynamics are not based on cancer stem cells, but on their progeny. The cells proliferate if space is available or become quiescent, otherwise. The tumours grow initially exponentially, but only up to a certain size, depending on the life span of the non-stem progeny. Tumour size then remains pseudostable over the long term, subject to short-term oscillations due to cancer cells dying and previously quiescent cells becoming active again (case for *ρ*_max_=10, *μ*=0 and *α*=0% shown in Figure 3). Depending on *ρ*_max_, it may take a long time until the stem cells become exposed to space again and potentially divide symmetrically to produce another cancer stem cell. Tumours with non-stem cell proliferation capacities, *ρ*_max_=15 and *ρ*_max_=20, and disabled migration and spontaneous cell death remain non-growing for the simulated *t*=20 years with average sizes of 213±36 cells and 430±31 cells at the end of the simulation, respectively (*n*=40 simulations) (Figure 4A–C). With a cancer cell migration of five cell widths per day (*μ*=5), the cluster can expand as more cells have space available to proliferate, resulting in tumours with average sizes of 1641±683 cells (for *ρ*_max_=15) and 1916±351 cells (for *ρ*_max_=20). However, even in this case there is insufficient opportunity for symmetric stem cell divisions to take place, and no appreciable tumour growth takes place over the 20 years simulated (Figure 4c). If the tumour cells have some migratory ability (*μ*=5) and also have progeny with a low proliferation capacity (*ρ*_max_=10), frequent symmetric stem cell divisions can be observed resulting in a stem cell pool of 239 cells and an average tumour size of 87 506 cells after about 16 simulated years. A further increase in migration (*μ*=15) for *ρ*_max_=10 results in a similar tumour size of 88 237 cells (131 cancer stem cells) in as short as 4 years. On the other hand, tumours with a cellular migration rate, *μ*=15, and progeny proliferation capacities, *ρ*_max_=15 and *ρ*_max_=20, will only feature on average 4.7 and 1.2 stem cells, and produce final tumour sizes of 10 051 and 4393 cells after *t*=20 years, respectively. Interestingly, the combination of a high migration rate (*μ*=15) and a low progeny proliferation capacity leads to a qualitative change in the behaviour of the population. These migrations into lower-density space favour the local expansion of stem and non-stem cells alike, but because stem cells may now engage in more frequent symmetric divisions, further cycles of stem cell seeding and colony expansions are enabled. In aggregate, the growing tumour mass consists of conglomerates of independent cell clusters developing in the vicinity of each other – the product of a continual ‘self-metastasis’ process that has recently been proposed to drive tumour growth (Norton, 2005; Norton and Massague, 2006).

Unexpectedly, our studies show that tumour progression is promoted when the non-stem progeny are short-lived. To test the limits of this counterintuitive effect, we introduced spontaneous cell death at *α*=5% per day to see how the system would respond to the further availability of space at the expense of a portion of the non-stem progeny. Incredibly, tumours with short-lived progeny (*ρ*_max_=10), migration rate, *μ*=15, and spontaneous cell death, *α*=5%, expand dramatically and populate almost the entire domain (100 000 cells) with 272 stem cells on average in just over 3 years, that is, *t*=38 months (Figure 4). At this time, tumours with higher proliferation capacities (*ρ*_max_=15 and *ρ*_max_=20) attain sizes of only 15 701 cells (with 10 stem cells), and 6542 cells (with 3 stem cells), respectively. Without migration, symmetric stem cell division can be seen, but the tumour cannot expand. The results collectively show that migratory ability dominates the kinetics. If the migration rate is insufficient, no values for proliferation and cell death can overcome tumour inhibition. By contrast, if migration is high, tumour size advances for a broad range of proliferation capacities and cell death rates, with both exerting counterintuitive long-term effects on the migration-enabled growth. Substantial paradoxical increases in ultimate tumour size attained are observed as α increases from 0 to 5% (when *ρ*_max_=10, 15 or 20), and as *ρ*_max_ decreases from 20 to 10 (when *α*=0 or 5%).

**Figure 5:**
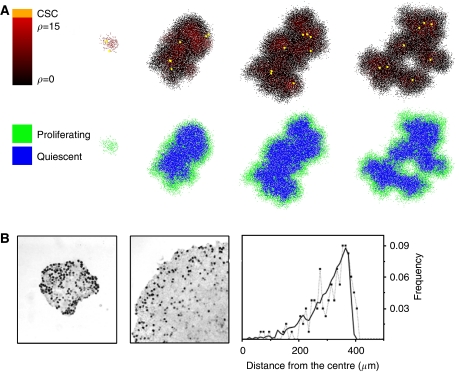
(**A**) Spatiotemporal evolution of the self-metastatic tumour population at different time points. Shown is the proliferative capacity and the proliferative state distribution. Proliferation mainly occurs on the tumour boundary, and cells with high proliferative capacity are located in the quiescent core. (**B**) Cell proliferation analysis with BrdU staining in a HCT116 cell population growing in culture at 7 and 13 days and radial distribution of BrdU positive cell frequency (reproduced by kind permission of Springer, from Galle *et al*, 2009).

The phenomenon of self-metastasis is largely determined by the migration rate, but is assisted by a low proliferation capacity. Simulations show that cells in the tumour core are predominantly quiescent, and cell proliferation occurs almost exclusively on the tumour boundary. Figure 5A shows the spatiotemporal evolution of a tumour exhibiting the hallmark of self-metastasis – cluster formation at the tumour periphery fuelled by stem cells that have migrated away from the main mass. Potent cells along with the cancer stem cells are located in the core of individual clusters, and the outer rim consists of cells with low remaining proliferative capacity, *ρ*. Visualisation of the proliferative state shows that proliferation occurs predominantly on the outer rim of the tumour, and the cells with high proliferative capacity reside in the quiescent core. In a study of the proliferation distribution of HCT116 cells in culture, Galle *et al* (2009) showed that proliferation indeed occurs mainly on the the tumour boundary. In line with our simulations, proliferation frequency decreases towards the tumour core, where proliferation is almost completely absent. Similar results were reported by Brú *et al* (2003), who have attributed intra-tumoural quiescence to the pressure exerted on the cells through lack of space. Cells proliferate if they have enough space, but become inhibited with increasing cell density (Figure 5B). An exponential decrease in mitotic cell distribution from the tumour boundary to the core is observed, both *in vitro* using BrdU staining in a Hela cell line and *in vivo* using a Ki-67 marker in colon adenocarcinoma. Remarkably, in the *in vivo* study, the decrease of proliferation was not because of hypoxia as blood vessels were present.

**Figure 6:**
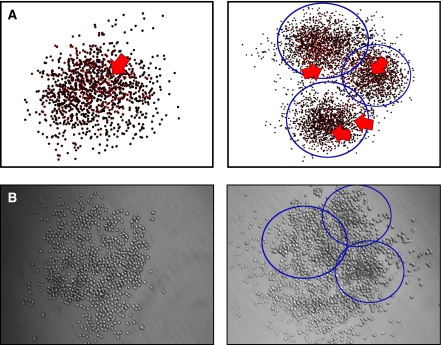
(**A**) All the common features of cell growth, including initial formation of gaps in tumour clones (left) and growth by self-seeding of clones (right), can readily be explained by basic cell kinetics. The arrows indicate locations of cancer stem cells in the simulation with parameters, *ρ*=10, *α*=5% and *p*_s_=1%. (**B**) Shown for comparison are tumourigenically transformed murine lung fibroblasts displaying migration-dependent clusterings arising from a single-plated cell per well.

**Figure 7:**
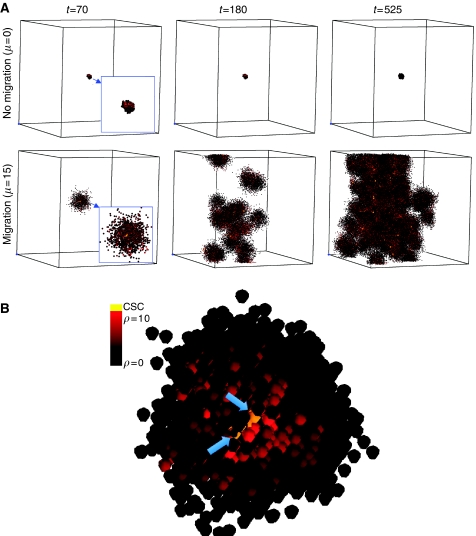
Three-dimensional simulations elucidating the pivotal role of migration in tumour growth and progression. (**A**) Without migration (*μ*=0, top row), no cells can be shed from the tumour to form foci of micrometastases and the tumour remains dormant. Cancer cell migration (*μ*=15, bottom row) is necessary to exhibit tumour growth and progression over time (*t*=70, 180, 525 days, left to right) through the formation of self-metastases. (**B**) High-resolution visualisation of a representative three-dimensional tumour cluster after the cancer stem cell (CSC) (yellow) has divided (arrows). Both stem cells are in the core of the tumour cluster with a radial proliferation capacity gradient.

We further find that all the common features of cell growth, including initial formation of gaps in tumour clones, the filling of those gaps and growth by self-seeding of clones (Figure 6) are all readily explained by the same basic kinetics as can account for migration- and space-limited growth. Moreover, the importance of migration in these several roles is seen not to be a two-dimensional artifact, but persists and is even facilitated in three dimensions owing to the increased opportunity for a given cell to move to an adjacent lattice point (Figure 7). In this process, it is noted that both stem and non-stem cells initiate new clones, although it is only the stem-cell-initiated clones that persist.

## Discussion

In this study, we developed an individual cell agent-based model of early tumour growth on the basis of the dynamics of cancer stem cells and their progeny tumour cells with limited proliferation capacity. In line with earlier studies, our model shows that increased migration drives the tumour progression (Betteridge *et al*, 2006), but in contrast to another model attributing similar results to patterned environments (Mansury *et al*, 2002), our study shows that complex dynamics and spatial agglomeration of tumour clusters can occur even when the environment is homogeneous and migration undirected. Although clusters can arise from gradated environments, this dependence should not be taken as absolute. Rather, we show that tumour growth coincides with cluster formation, and that such clusters can form in the absence of gradients. Although these results should not be taken to mean that effects due to larger-scale tissue-level dynamics, for example, angiogenesis and immunity, are just elaborations of effects already accounted for by cell-level kinetics, they do alert us to the possibility of effect overlaps that may confound causal inference.

Single-cell-based experimental models and their computational counterparts have proven to be a powerful tool to mimic, compare and explain cell behaviour *in vitro*, especially in monolayer cultures and three-dimensional spheroids (Drasdo and Höhme, 2005). As confirmed *in silico*, tumour growth *in vitro* is well described by an initially exponential growth followed by a sub-exponential phase, because of inhibition of proliferation in the tumour interior. Proliferation occurs predominantly on the outer rim, with cells in the interior being quiescent because of space inhibition or becoming necrotic because of oxygen or glucose deprivation. Experiments have also shown proliferation age to be higher near the outer rim in the sub-exponential populations. Basanta *et al* (2008) have described the interplay of proliferative and motile phenotypes in a developing tumour using game theory, applying fitness cost tradeoffs between the two phenotypes. Motile cells migrate away from cell clusters so that when they become proliferative, they promote tumour expansion. With increasing costs of motility, however, the fraction of proliferative cells increases and interestingly reduces tumour invasion. Although their approach was different, their results and ours both suggest that cell movement may be a critical determinant of tumour growth. In other computer models, tumour invasion was characterised by the transformation of a spherical tumour into fingering morphologies (Anderson, 2005). Invasive morphologies emerge upon harsh microenvironmental conditions, and the dynamical changes are driven by extreme changes in nutrient supply during tumour development (Anderson *et al*, 2009). They found that in a homogeneous environment without haptotactic and chemotactic gradients to direct cell migration, the tumour morphology is persistently radially symmetric. Our results go on to show that fingering morphologies may also happen in homogeneous environments. One rationalisation for this *vis a vis* those studies may be that our assumption of a small stem cell fraction may effectively allow for a source of heterogeneity *within* the population that substitutes for the lack of milieu heterogeneity considered by Anderson *et al* to be necessary for expression of this phenotype.

**Figure 8:**
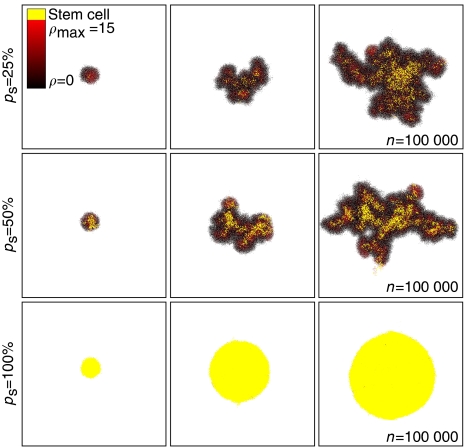
Symmetric division rate and tumour morphology in simulations of tumour growth up to 100 000 cells. Symmetric division rates of *p*_s_=25% and *p*_s_=50% result in self-metastatic growth comparable with *p*_s_=1% (Figures 4, 5, 6 and 7). The stem cell pool in each tumour cluster increases with increasing *p*_s_ and cancer stem cells are enriched in the tumour core. A tumour composed of only stem cells, that is, *p*_s_=100%, features a radially symmetric morphology. Parameters are *ρ*_max_=15, *μ*=15 and *α*=1.

Our study has focused on three critical intrinsic mechanisms that determine individual cell behaviour – proliferation, migration and cell death. Computer simulations of the model indicate that only certain parameter regions of the three-dimensional parameter space (proliferation capacity, migration rate and cell death rate) are consistent with malignant tumour growth, and that the dependence can be complicated. Non-monotonic dependencies were noted for both proliferation capacity and cell death rates for a given migration rate. In our model, each stem cell is only capable of producing a tumour cluster of a limited size, unless progeny proliferation capacity, spontaneous cell death and migration rate are such as to provide space for the reproduction of stem cells and growth of their non-stem-cell progeny. Increasing the cell death rate or decreasing the proliferation capacity within certain ranges counterintuitively helps to liberate stem cells that need space to proliferate. In addition, the main consequence of increasing the stem cell renewal probability, *p*_s_, from 1% assumed throughout is only a slight increase in the degree of branching of the tumour (Figure 8), up to percentages as high as 50%. Still higher percentages lead to progressively decreasing branching, until at 100%, a smooth circular tumour results (Anderson, 2005; Drasdo and Höhme, 2005). It is concluded that, although the branching morphology relies on the presence of stem cells, the phenotype will disappear if we adopt the common presumption that all cancer cells are cancer stem cells.

One consistent feature of malignant tumour growth is the ability of cancer stem cells to migrate into less dense space to form separate tumour clusters. The aggregate growth of all tumour clusters results in population dynamics that are widely observed – initial exponential growth, followed by a linear growth and a final plateau phase. The observed tumour population dynamics support a recent hypothesis of Norton and coworkers (Norton, 2005; Norton and Massague, 2006) that a tumour grows by a process of self-metastasis – the shedding of stem cells into the tumour periphery that expand and merge to advance the tumour boundary. Cell migration is necessary for the self-seeding process, and without migration, the tumour will be forced to remain dormant (Figure 4).

More broadly, the dependence of stem cell kinetics on cluster formation, made possible by focal separation, may inform our understanding of how stem cell prevalence and distribution relate to tumour growth overall. For cancer, gaining an improved understanding of how tumours can be driven by independently growing clusters governed by the rules of proliferation, cell death and migration may offer new insights into potential treatment strategies. As observed by Norton, current anti-proliferative cancer drugs have proven to shrink tumours, but fail to provide actual cures (Norton and Massague, 2006). Our model suggests that discouraging migration might provide an alternative means of cancer suppression. Importantly, the results suggest that anti-mitotic treatments alone, despite killing cancer cells, may actually promote tumour progression if eradication of cancer stem cells cannot be achieved (Michor *et al*, 2005; Dingli and Michor, 2006; Yang and Wechsler-Reya, 2007). Alternatively, in line with observations of Basanta *et al* (2008), discouraging migration may be an important new means of cancer suppression. How this would be facilitated using agents that rely on possibly counterproductive targeting is not straightforward. On the other hand, by iteratively refining agent-based models that track population-level responses to multimodality approaches, strategies that provide a more favourable long-term treatment response can be expected.
